# 53 Nakajo-Nishimura syndrome: the first African case

**DOI:** 10.1093/rheumatology/keac496.049

**Published:** 2022-10-07

**Authors:** Nacif Eddine Ghodbane, Ali Mecibah, Assia Haddouche, Halima Zerguine, Zineb Akakba, Samy Slimani

**Affiliations:** 1 Department of Otorhinolaryngology Head and Neck Surgery EPH Houas Salah ORL Clinic, allées Benboulaid Batna, Algeria; 2 Department of Otorhinolaryngology, Head and Neck Surgery EPH EPH Houas Salah ORL Clinic, allées Benboulaid Batna, Algeria; 3 University of Algiers 3, Algeria; 4 Department of Pediatrics CHU Benflis Touhami, allées Mohamed boudiaf, Batna, Algeria; 5 Department of Cardiology CHU mustapha pacha, place du 1er mai 1945 Sidi M’hamed, Algiers, Algeria; 6 Rhumatology Clinic, 81 cité des 500 logements, Batna, Algeria

**Keywords:** Nakajo-Nishimura syndrome, hereditary, Africa, Algeria, chronic atypical neutrophilic dermatosis with lipodystrophy and elevated temperature

## Abstract

Nakajo-Nishimura syndrome is a hereditary autoinflammatory disorder caused by an autosomal recessive homozygous mutation of the PSMB8 gene, which encodes the immunoproteasome subunit beta 5i. The clinical manifestations of NNS are mainly pernio-like skin rashes, nodular erythema, lipodystrophy, clubbed fingers, remittent fever, hepatosplenomegaly, and basal ganglia calcifications. Here we are reporting a case of NNS in an 11-year-old girl, who lives in eastern Algeria, born from a first-degree consanguineous marriage, she presented with erythematous patches on her face and her back, nodular erythema on her neck, swollen and painful fingers with acrocyanosis and recurrent fever that mainly occurred in cold weather. The patient received long-term treatment with low-dose glucocorticoids, along with immunomodulatory drugs (hydroxychloroquine with methotrexate), partial improvement clinically and biologically was observed. Colchicine was added to her treatment, with increased prednisone doses when she recently developed an AA amyloidosis. Our patient was diagnosed clinically with NNS because she exhibited six of the eight characteristics. To the best of our knowledge, this is the first case of NNS in Africa.

